# Correlation between Colon Polyps and Metabolic Syndrome and HP Infection Status

**DOI:** 10.1155/2019/3916154

**Published:** 2019-06-04

**Authors:** Lijuan Huang, Lihong Wu, Qiaohua Qiao, Lizheng Fang

**Affiliations:** Department of General Practice, Sir Run Run Shaw Hospital, Zhejiang University School of Medicine, Hangzhou, Zhejiang Province, China

## Abstract

**Background:**

This study investigated the relationships among the characteristics of colon polyps and potential risk factors, including metabolic condition, CEA level, uric acid level, and *Helicobacter pylori* (Hp) infection status.

**Method:**

Clinical data from patients who received colonoscopy were collected and analyzed, including patients' gender, age, polyp pathology, metabolic syndrome (MS) status, CEA level, uric acid level, and Hp infection status. Patients were divided into a polyp group and a control group based on whether they presented with colon polyps. Then, clinical data were compared between the two groups to identify any differences between the groups and their relationships to colon polyps.

**Result:**

Compared with the control group, the polyp group had significant differences in patient gender, body mass index (BMI), waistline, blood pressure, fasting blood glucose level, blood lipid level, and uric acid level (*p* < 0.05), but there were no significant differences in LDL and CEA levels (*p* > 0.05). Patients with MS or a uric acid level > 340 mg/dl had a greater tendency to develop colon polyps but this was not statistically significant.

**Conclusion:**

The incidence of colon polyps may be associated with MS and uric acid levels, but further studies are warranted to confirm this conclusion.

## 1. Introduction

Colon polyps, especially adenomatous polyps, have been widely regarded as precancerous lesions that are caused by various pathogenic factors, including heredity, inappropriate diet habits, and infections [1]. Among these pathogenic factors, *Helicobacter pylori* (Hp) infection plays an important role. HP infection has been reported to be associated with the pathogenesis of colon polyps [2]. Data have shown that the incidence of colon polyps has dramatically increased in the past 20 years in the Asia-Pacific area, which may have been associated with a high-fat, high-protein diet and a lack of physical exercise [3, 4]. There have also been multiple studies showing the relationship between colon polyps and metabolic syndrome- (MS-) associated indexes, including blood lipid level, glucose level, and BMI [3, 5–7]. In recent years, the detection rate of colon polyps has grown due to the increasing use of colonoscopy, and it has been reported that more than 85% of all colon cancer cases transform from colon polyps [8]. However, the association between the pathology and site of colon polyps and HP infection is poorly investigated. Besides, considering the metabolic factors may influence the formation of colon polyps, we discussed whether uric acid levels and MS status could serve as risk factors for the occurrence of colon polyp. CEA level was reported as a marker for the occurrence of colon polyps and was examined in our study [9].

## 2. Methods

Patients were identified from Sir Run Run Shaw Hospital, Zhejiang University, from Jan 2010 to May 2014. The included patients should have undergone gastroscopy, colonoscopy, C13 breath tests, and routine blood tests. Patients presenting colon polyps were classified as the polyp group, and those who did not present with colon polyps were classified as the control group. A C13 breath test was employed to test for HP infection, and those with positive results were considered Hp positive. Nearly all detected polyps were removed and colon polyp samples were processed, histological slices were made, and HE staining was used for pathologic diagnosis. Pathological classification of colon polyps included inflammatory polyp (I), proliferative polyp (P), adenoma (A), and cancer (C); sites of colon polyps included the left colon (L) (including the rectum and sigmoid and descending colon), transverse colon (T) (including splenic flexure, transverse colon, and hepatic flexure), and right colon (R) (including the ascending colon and ileocecal junction). Polyp size was assessed according to the Yamada standard and classified as Yamada type I, II, III, or IV; the number of polyps was classified as single or multiple. Our study was performed after obtaining informed consent from the patients and approval from the Ethics Committee of Sir Run Run Shaw Hospital, Zhejiang University.

Patients with chronic gastritis, digestive ulcers, and gastric cancer and who underwent previous gastric surgery were excluded by gastroscopy. Patients who underwent Hp eradication therapy or those with ulcerative colitis, Crohn's disease, or familial adenomatous polyps were also excluded.

SPSS 19.0 was employed for statistical analysis, and quantitative data were expressed in the form of (*X* ± *S*). A *t*-test was used to compare the differences. Enumeration data were expressed as *n* (%). The chi-square test was used to test the difference and *p* < 0.05 was accepted as statistically significant. Nonconditional logistic regression was expressed using the odds ratio and a 95% confidence interval.

## 3. Results

### 3.1. Baseline Characteristics of Patients from the Polyp Group and the Control Group

In this study, 159 patients were enrolled in the polyp group and 334 patients were enrolled in the control group. The average age of the patients was 50.10 ± 8.29 years for the polyp group and 46.22 ± 8.13 years for the control group; the difference was significant (*p* < 0.0001). Significant differences were also found in gender, BMI, waistline, blood pressure, fasting blood glucose level, blood lipid level, and uric acid level (*p* < 0.05), but no significant difference in LDL and CEA was detected between the two groups (*p* > 0.05) (Table 1).

### 3.2. Relationship between Hp Infection/MS and Colon Polyps

For patients with multiple colon polyps, the largest polyp was assessed for location and Yamada classification, and the most advanced pathological type of all polyps was taken as the pathological status of the patient. Comparing the H. pylori- group and the H. pylori+ group, there were no significant differences in polyp number, site, and pathology (*p* > 0.05). In the subgroup analysis, polyp patients with MS had significantly more polyps than those without MS, and no significant difference was detected in polyp size, location, and pathological type in the subgroups described previously (Tables 2(a) and 2(b)).

### 3.3. MS Status, CEA Level, Uric Acid Level, and Hp Infection Status in Patients with Colon Polyps

Within the 157 colon polyp patients, 63 had metabolic syndrome (MS) (40.13%), whereas 73 of the 334 (22.26%) patients in the control group had MS; the difference was significant (*p* = 0.0023). For all enrolled patients, subgroup analysis showed that patients with MS had a significantly greater chance of developing colon polyps (OR = 2.16, 95% CI = 1.32 − 3.54).

In this study, we also found that the polyp group and the control group had no significant difference in CEA level, uric acid level, and Hp infection status. If we divide all enrolled patients by the median uric acid level (340 mg/dl) into a high uric acid level group (≥340 mg/dl) and a low uric acid level group (<340 mg/dl), the relative risk of developing colon polyps in the former group was 0.83 times higher than that in the latter group (95% CI = 0.52 − 1.3). Similarly, for all patients, Hp-positive patients had a relative risk of developing polyps that was 1.15 times higher than HP-negative patients (95% CI = 0.76 − 1.74) (Table 3).

### 3.4. Interactive Variant Analysis of All of the Related Factors

Interactive variant analysis was used to study the interrelationship among factors, including MS status, CEA level, uric acid level, and Hp infection status. The results revealed that the median value of the CEA level of the enrolled patients was 1.43, and we defined those with CEA < 1.43 as low CEA and those with CEA ≥ 1.43 as high CEA. Patients with MS had a higher risk of developing colon polyps independent of their CEA level, but as patients' CEA level increased, the risk of developing colon polyps also increased (OR = 2.58, 95% CI = 1.29 − 5.17); however, these two factors had no interactive effect (OR = 1.22, 95% CI = 0.51 − 2.90).

Compared with patients who had high uric acid level and did not have MS, patients with MS had a higher risk of developing colon polyps independent of their uric acid level; however, this risk escalated as patients' uric acid level decreased (OR = 2.58, 95% CI = 1.13 − 5.89). These above two factors also had no interactive effect (OR = 1.35, 95% CI = 0.53 − 3.44) (Table 4).

Overall, no significant interactive effects existed among MS, CEA level, uric acid level, and Hp infection state.

## 4. Discussion

Recently, there have been multiple studies concerning Hp infection and colon polyps, and the results showed that patients with adenomatous polyposis coli (APC) had a high incidence of Hp infection; therefore, it has been hypothesized that Hp infection could increase the risk of adenoma and adenocarcinoma in the colon and rectum, and their relationship was also demonstrated by the mucosal proliferation/APC/adenocarcinoma axis [10–12]. Zumkeller et al. analyzed the literature from 1991 to 2002 in a meta-analysis and showed that Hp infection might be associated with the pathogenesis of colon adenoma and adenocarcinoma, with an OR of 1.4 (95% CI: 1.1-1.8) [13]. Oh et al. found that gastric cancer patients had a higher risk of colon cancer [14]. Additionally, Soylu et al. found Hp in samples of colon adenoma by immunohistochemistry. All of these studies suggest a potential relationship between Hp infection and colon cancer. There have also been studies that have presented conflicting results. For example, in a prospective study, the Hp infection rate was not significantly different between patients with colon polyps/cancer and a normal cohort, which did not support the hypothesis that the polyps were relevant to the formation of cancer [11, 15]. Additionally, Abbass et al. proposed that the incidence of colon polyp/cancer was not significantly different in Hp-positive and Hp-negative patients [16].

Several meta-analyses of the literature from the Western world showed that Hp infection had a certain relationship with the pathogenesis of colon polyps and that Hp infection increased the risk of colorectal adenoma and adenocarcinoma; however, the meta-analysis drew no concrete conclusion [11, 17]. Several studies have investigated the relationship between Hp infection and colon polyps in the Chinese population [12]. Lin et al. launched a cross-sectional study in Taiwan province and found that MS patients with Hp infection were more susceptible to colorectal adenoma [18].

The mechanisms of how Hp infection could induce colon polyps and carcinomas are still unclear, and research has shown that gastrin and cyclooxygenase-2 (COX-2) were involved; gastrin can regulate gastric acid secretion, promote the proliferation of gastric mucosa, and promote the proliferation and metastasis of gastric tumor cells [19, 20]. COX is the rate-limiting enzyme of prostaglandin synthesis, and under pathological conditions, COX-2 promotes tumor cell proliferation, inhibits cell apoptosis, promotes the release of angiopoietin, inhibits endothelial apoptosis, and activates MMPs (matrix metalloproteinases), thus inducing tumor angiogenesis. It has been suggested that aspirin can reduce the incidence of colon adenomas and individuals who took COX-2 inhibitors had a lower incidence of colon cancer than those who did not [21]. However, there were also different conflicting data, and Selgrad et al. found that gastrin did not increase patients' risk of colorectal cancer [22]. In summary, the role of Hp in the pathogenesis of colon polyps is still unclear.

Besides, metabolic factors were also reported as risk factors for polyp occurrence. A cohort study involving 2244 patients indicated that visceral adipose tissue was dose-dependently associated with colorectal adenoma [23]. Similarly, abdominal obesity, hypertension, and a high HbA1c% increase the risk for polyps [24]. Consistent with previous reports, we found that uric acid and MS index were also strongly associated with colon polyp. Uric acid is an important damage-associated molecular pattern (PAMP) and exerts strong proinflammation effect [25]. Monosodium urate crystals stimulate inflammatory response by activation of toll-like receptor 4, which strongly promote leukocyte to produce proinflammatory cytokines. The proinflammatory factors were known to play a critical role in regulating neoplasm formation and cancer progress [26]. Therefore, reducing serum uric acid level may decrease level of systemic inflammation and break polyp-cancer transformation.

In this study, we found that the risk of colon polyps escalated as patients' age increased and that compared to women men were more likely to have colon polyps. We also found that MS was a risk factor for colon polyps. Within MS patients, colon polyp risk escalated as CEA level increased, but the risk declined as uric acid level increased. Hp infection may increase the risk of colon polyps. This study was retrospective and had a limited sample size. Further prospective studies with a larger cohort of patients are needed to verify our hypothesis.

## Figures and Tables

**Table 1:** Baseline characteristics of patients in the polyp group and the control group.

Variables	Controls (*N* = 334)	Cases (*N* = 159)	*p*
Gender (male)	239 (71.56)	138 (86.79)	0.0002
Family history of cancer (yes)	77 (23.05)	41 (25.79)	0.5063
Age	46.22 ± 8.13	50.10 ± 8.29	<0.0001
BMI	24.29 ± 3.06	25.17 ± 3.17	0.0032
Waist^a^	85.41 ± 9.33	88.94 ± 10.02	0.0002
SBP	116.17 ± 12.33	122.85 ± 13.76	<0.0001
DBP	70.24 ± 9.11	75.25 ± 10.48	<0.0001
FPG	4.85 (4.50, 5.20)	5.07 (4.72, 5.44)	<0.0001
TG	1.39 (0.91, 2.11)	1.72 (1.17, 2.42)	0.0013
TC	4.69 ± 0.93	4.87 ± 1.01	0.0441
HDL	1.15 ± 0.28	1.07 ± 0.24	0.0032
LDL^b^	2.64 ± 0.83	2.80 ± 0.88	0.0535
CEA	1.43 (0.82, 2.13)	1.64 (0.82, 2.52)	0.0609
UA	342.40 ± 95.78	366.03 ± 80.23	0.0044

^∗^Categorial variable, *n* (%); continuous variable, mean ± SD/median (p25, p75). ^a^Data for two cases and six controls were unknown. ^b^Data for 12 cases were unknown.

**Table:** (a) Polyp parameters and Hp infection status

	H. pylori+	H. pylori-	*p*
No. of polyps			0.9085
None	208 (62.28)	126 (37.72)	
Single	66 (60.55)	43 (39.45)	
Multiple	32 (64.00)	18 (36.00)	
Polyp size			0.3518
I	67 (65.05)	36 (34.95)	
II	12 (48.00)	13 (52.00)	
III	14 (66.67)	7 (33.33)	
IV	5 (50.00)	5 (50.00)	
Polyp location			0.5707
L	74 (63.79)	42 (36.21)	
R	12 (52.17)	11 (47.83)	
T	12 (60.00)	8 (40.00)	
Polyp pathology^a^			0.6865
A	57 (58.76)	40 (41.24)	
P	17 (60.71)	11 (39.29)	
I	23 (69.70)	10 (30.30)	
C	1 (100.00)	0 (0.00)	

^∗^^a^Fisher's exact test.

**Table:** (b) Polyp parameters and MS status

Polyp	MS		*p*
	No	Yes	
No. of polyps			0.0002
None	255 (77.74)	73 (22.26)	
Single	66 (61.11)	42 (38.89)	
Multiple	28 (57.14)	21 (42.86)	
Polyp size			0.2315
I	63 (61.76)	39 (38.24)	
II	14 (58.33)	10 (42.67)	
III	14 (66.67)	7 (33.33)	
IV	3 (30.00)	7 (70.00)	
Polyp location			0.5755
L	69 (60.53)	45 (39.47)	
R	15 (65.22)	8 (34.78)	
T	10 (50.00)	10 (50.00)	
Polyp pathology^a^			0.6926
A	59 (61.46)	37 (38.54)	
P	17 (60.71)	11 (39.29)	
I	18 (56.25)	14 (43.75)	
C	0 (0.00)	1 (100.00)	

^∗^^a^Fisher's exact test.

**Table 3:** MS, CEA, UC, and Hp infection status in the polyp group and the control group.

Variables	Controls (*N* = 334)	Cases (*N* = 159)	OR (95% CI)	*p*
MS				
No	255 (77.74)	94 (59.87)	1.00	—
Yes	73 (22.26)	63 (40.13)	2.16 (1.32, 3.54)	0.0023
CEA				
<1.43	167 (50.00)	67 (42.14)	1.00	—
≥1.43	167 (50.00))	92 (57.86)	1.06 (0.71, 1.58)	0.7867
UA				
<340	167 (50.00)	70 (44.03)	1.00	—
≥340	167 (50.00)	89 (55.97)	0.83 (0.52, 1.30)	0.4094
H. pylori				
Negative	208 (62.28)	98 (61.64)	1.00	—
Positive	126 (37.72)	61 (38.36)	1.15 (0.76, 1.74)	0.4990

^∗^OR adjusted for age, gender, BMI, and family history of cancer.

**Table 4:** Interactive analysis among MS, CEA, UC, and Hp infection status.

Variable 1	Variable 2	Controls (*N* = 334)	Cases (*N* = 159)	OR (95% CI)	*p*
*MS*	*CEA*				
No	<1.43	125 (38.11)	37 (23.57)	1.00	—
No	≥1.43	130 (39.63)	57 (36.31)	1.06 (0.64, 1.78)	0.8114
Yes	<1.43	39 (11.89)	29 (18.47)	1.99 (1.02, 3.86)	0.0432
Yes	≥1.43	34 (10.37)	34 (21.66)	2.58 (1.29, 5.17)	0.0075
Interaction OR				1.22 (0.51, 2.90)	0.6519
*MS*	*UA*				
No	≥340	114 (34.76)	43 (27.39)	1.00	—
No	<340	141 (42.99)	51 (32.48)	1.41 (0.83, 2.42)	0.2067
Yes	≥340	52 (15.85)	46 (29.30)	2.46 (1.38, 4.40)	0.0024
Yes	<340	21 (6.40)	17 (10.83)	2.58 (1.13, 5.89)	0.0242
Interaction OR				1.35 (0.53, 3.44)	0.5330
*MS*	*H. pylori*				
No	Negative	163 (49.70)	61 (38.85)	1.00	—
No	Positive	92 (28.05)	33 (21.02)	1.13 (0.67, 1.89)	0.6518
Yes	Negative	42 (12.80)	36 (22.93)	2.15 (1.18, 3.91)	0.0124
Yes	Positive	31 (9.45)	27 (17.20)	2.41 (1.23, 4.73)	0.0106
Interaction OR				1.00 (0.42, 2.38)	0.9932
*H. pylori*	*CEA*				
Negative	<1.43	102 (30.54)	43 (27.04)	1.00	—
Negative	≥1.43	106 (31.74)	55 (34.59)	0.94 (0.57, 1.57)	0.8230
Positive	<1.43	65 (19.46)	24 (15.09)	0.98 (0.53, 1.81)	0.9438
Positive	≥1.43	61 (18.26)	37 (23.27)	1.24 (0.70, 2.18)	0.4642
Interaction OR				1.34 (0.59, 3.05)	0.4853
*H. pylori*	*UA*				
Negative	≥340	102 (30.54)	56 (35.22)	1.00	—
Negative	<340	106 (31.74)	42 (26.42)	1.04 (0.60, 1.79)	0.8876
Positive	≥340	65 (19.46)	33 (20.75)	0.96 (0.55, 1.65)	0.8684
Positive	<340	61 (18.26)	28 (17.61)	1.55 (0.81, 2.93)	0.1832
Interaction OR				0.64 (0.28, 1.47)	0.2935
*CEA*	*UA*				
<1.43	≥340	72 (21.56)	36 (22.64)	1.00	—
<1.43	<340	95 (28.44)	31 (19.50)	1.03 (0.54, 1.95)	0.9338
≥1.43	≥340	95 (28.44)	53 (33.33)	0.93 (0.54, 1.60)	0.7969
≥1.43	<340	72 (21.56)	39 (24.53)	1.30 (0.70, 2.42)	0.4133
Interaction OR				0.74 (0.33, 1.65)	0.4576

^∗^OR adjusted for age, gender, BMI, and family history of cancer. *CEA (1.43) and UA (340)* were determined by the corresponding median value of enrolled patients. Interaction OR: interactive effect of the multiplicative model.

## Data Availability

The data used to support the findings of this study are available from the corresponding author upon request.
